# What we should consider to facilitate recovery of the hematological profile in all patients after pancreaticoduodenectomy: the role of preoperative intravenous iron treatment

**DOI:** 10.1186/s12893-023-02217-x

**Published:** 2023-10-12

**Authors:** Eun Young Kim, Sung Eun Park, Tae Ho Hong

**Affiliations:** 1 Division of Trauma and Surgical Critical Care, Department of Surgery, Seoul St. Mary’s Hospital, College of Medicine, The Catholic University of Korea, Seoul, Republic of Korea; 2 Division of Hepato-biliary and Pancreas Surgery, Department of Surgery, Seoul St. Mary’s Hospital, College of Medicine, The Catholic University of Korea, 222, Banpo-daero, Seocho-gu, Seoul, 06591 Republic of Korea

**Keywords:** Intravenous iron, Iron-deficiency anemia, Pancreaticoduodenectomy

## Abstract

**Background:**

In pancreaticoduodenectomy (PD), the duodenum and upper jejunum responsible for iron absorption are removed, which can lead to massive hemorrhage during surgery and cause iron deficiency anemia after PD. The aim of this study was to evaluate overall changes in hematologic profiles of patients who underwent pancreaticoduodenectomy. Effect of preoperative intravenous iron treatment on recovery of anemia after surgery was also investigated.

**Methods:**

From March 2021 to December 2021, patients who underwent curative PD at our institution due to periampullary lesions were enrolled. They were divided into two groups according to whether or not iron was administered before surgery. In the IV iron group, all patients had been routinely administered with 1000 mg of ferric carboxymaltose intravenously once about 3–7 days before the operation day. Contrarily, patients in the control group did not receive intravenous iron before PD. Changes in hematological profile were measured preoperatively and at 5, 14, and 30 days postoperatively. Clinical results of the two groups were compared and analyzed. Additionally, a subgroup analysis was performed for selected non-anemic patients who had preoperative hemoglobin level of 12.0 g/dl or higher to compare changes in hematologic profiles between the two groups.

**Results:**

Thirty patients of the IV iron group and 34 patients of the control group were analyzed. Although no difference was observed in postoperative complications or mortality, hemoglobin and iron levels were recovered significantly faster at two weeks postoperatively in the IV iron group than in the control group. Iron levels were significantly higher in the IV iron group throughout the postoperative period. In subgroup analysis conducted for non-anemic patients, hemoglobin levels were recovered significantly faster and maintained higher in the IV iron group throughout the postoperative period, although baseline levels of hemoglobin were similar between the two groups. In addition, the length of intensive care unit stay was significantly shorter in the IV iron group than in the control group.

**Conclusions:**

Preoperative intravenous iron treatment might be effective in facilitating recovery of hematologic profiles of patients during the recovery period after PD regardless of the presence of preoperative anemia, thus preventing postoperative iron deficiency anemia.

**Supplementary Information:**

The online version contains supplementary material available at 10.1186/s12893-023-02217-x.

## Introduction

Iron deficiency anemia (IDA) is commonly observed in cancer patients [1]. It has multifactorial etiologies such as bleeding, insufficient iron absorption, and anemia related to malignancy or chronic disease, a cytokine-mediated disorder [2]. In case of cancer surgery, since it generally involves tissue manipulation for organ resection and radical lymph node dissection, systemic inflammation can occur after surgery. It can induce the main regulator of systemic iron hemostasis that impairs intestinal iron absorption, thus increasing the risk of IDA in patients who undergo cancer surgery [3, 4].

Pancreaticoduodenectomy (PD), a representative surgery for periampullary tumorous lesions, is a major operation usually accompanied by moderate bleeding and massive tissual injury that are predisposing factors of IDA. Moreover, PD has vulnerable points for IDA physiologically because it inevitably removes the duodenum which absorbs iron into the body. Thus, the function of iron absorption is impaired after PD. In addition, an insufficient oral intake for a relatively long period after PD is common due to complex anastomosis. Therefore, the risk of IDA after PD can be very high regardless of presence or absence of preoperative anemia. IDA is a well-known risk factor of postoperative morbidities and poor prognosis of patients after surgeries [1]. Allogenic transfusion to correct anemia can be associated with both infectious and noninfectious risks such as cancer recurrence, cardiac complication, and prolonged hospitalization [5]. Several studies have reported that diagnosing anemia before surgery and correcting it using intravenous (IV) iron preoperatively can improve postoperative prognosis of patients [5–8]. In the field of colorectal or cardiac surgery, this effect in people diagnosed with IDA before surgery has been reported. However, the standard pattern of changes in hematologic parameters after PD has not been reported. There is a lack of studies about the effect of preoperative iron injection on clinical outcome of PD.

Thus, the aim of this study was to evaluate changes in hematologic profiles throughout the entire period of treatment in patients who underwent PD due to periampullary lesions. Effects of preoperative iron treatment on correction of postoperative anemia and improvement of clinical outcomes within 30 days after this surgery were also evaluated.

## Methods

### Study design and patient enrollment

This observational study was approved and carefully monitored by our Institutional Review Board (IRB No. KC22RISI0417). From March 1, 2021 to December 31, 2021, patients admitted to our institution planning to undergo curative PD for periampullary lesions were enrolled. Periampullary lesion included pancreas cancer, ampulla of Vater cancer, cholangiocarcinoma of the distal common bile duct, and duodenal cancer depending on the location of the origin lesion, and all of these were included in the analysis. PD was performed by one surgeon specialized in hepato-biliary-pancreas surgery. In principle, we preferentially applied laparoscopic PD to patients, and performed open PD for some patients for whom laparoscopic approach was not possible due to severe adhesions from previous surgery. From August 1, 2021, patients had been routinely administered iron intravenously at the outpatient department visit about 3–7 days before the operation day regardless of their preoperative hemoglobin (Hb) levels. Contrarily, patients admitted from March 1, 2021 to July 31, 2021 did not receive intravenous iron treatment before the surgery. For analysis of the current study, we categorized participants into two groups: an IV iron group and a control group. Exclusion criteria were: (a) palliative or emergent surgery, (b) non-curable or metastatic disease status, (c) absence or insufficient data of essential laboratory values, (d) concomitant hematological disorders that might cause anemia, or (e) loss of follow-up. Patients who met the exclusion criteria were excluded. Contraindications for using intravenous iron were as follows: pregnant or lactation, age ≤ 18 years, history of severe asthma or infections, chronic renal failure, simultaneous oral iron or intravenous administration, or having any allergic reaction to iron [9, 10].

### Preoperative intravenous iron infusion

After the elective surgery was planned, patients in the IV iron group received the intravenous iron once about 3–7 days before the operation day, when usually patient last visit outpatient department prior to surgery that the last outpatient visit and surgery plan decision are made according to the policy of our institution. As previously reported in the study of Ionsescu et al., [5] as there is rapid and direct binding of IV iron to plasma transferrin, the erythropoietic effect increases about 5 times more than oral iron, and lasts for 7 days. The preparation used was 1000 mg of ferric carboxymaltose (Ferinject™, Vifor Pharma, Glattbrugg, Dwitzerland) that was diluted in 250 ml of 0.9% normal saline using an aseptic technique. It was intravenously infused over 15 min under the supervision of a clinician according to the manufacturer’s recommendation [6, 7, 11, 12]. Clinical monitoring was carefully conducted by a nurse, including vital signs or any signs of hypersensitivity.

### Data recruitment and clinical outcome assessment

All medical data and operative records were collected from patients and retrospectively reviewed. For all participants, laboratory data were routinely obtained preoperatively and on the 5th day, 14th day, and 30th day postoperatively. Laboratory values included hematologic profiles such as hemoglobin, iron, transferrin, and iron. For oncologic profiles, TNM cancer staging was defined based on pathologic reports using the American Joint Committee on Cancer Staging Manual, 8th edition [13]. Postoperative complications were reviewed and classified according to the *Clavien-Dindo* classification [14]. Complications of grade I and II were considered as minor complications, whereas grade III to V complications were defined as major complications. Detailed definition of complications was described in Appendix 2. Postoperative pancreatic fistula (POPF) was defined and classified as Grade A, B, or C according to the updated definition of the International Study Group in Pancreatic Surgery in 2016 [15]. In this study, we considered only Grade B and C POPF to be clinically significant and regarded as postoperative leakage. Wound complications was defined as superficial incisional, deep incisional, or organ-occupying soft tissue infection (SSI). A simple hematoma or seroma which did not require any treatment was not considered as a wound complication. Postoperative bleeding was defined as the requirement of transfusion of red blood cell within 72 h after the start of surgery. Postoperative mortality was defined as any mortality within 30 days of surgery or within the same hospitalization as the surgery. All patients underwent the same clinical protocol during hospitalization regardless of the diagnosis or type of surgery. They were given parenteral nutritional support on the first day after operation if their vital signs were stable. Oral feeding was resumed on postoperative day 3 unless there was any evidence of pancreas leakage. On postoperative day 5, patients were routinely evaluated by abdominal computed tomography scans to confirm the absence of pancreas leakage and any other intraoperative complications. Regarding red blood cell transfusion postoperatively, it was conducted according to the Joint United Kingdom Blood Transfusion and Tissue Transplantation Services Professional Advisory Committee guidelines for surgery [16]. Transfusion is recommended if hemoglobin levels are lower than 7.0-8.0 g/dL. However, the final decision of red blood cell transfusion was based on clinical conditions of the patient. Considerations for discharge were fine condition with oral analgesics and tolerable resumption of oral diet.

We analyzed and compared patterns of changes in hematologic profiles by measurement period (preoperatively, the 5th day, 14th day and 30th day postoperatively) and clinical outcomes between the IV iron group and the control group during the entire observational period. In addition, by applying the criteria used in several previous studies [17–19], patients with a preoperative Hb level of 12.0 g/dL or higher were selected and defined as preoperative non-anemic patients. We then performed a subgroup analysis comparing differences of changes in hematologic profiles between the IV iron group and the control group of preoperative non-anemic patients. The primary outcome of current study was the hematologic profiles after surgery, and the secondary outcome was the rate of postoperative morbidities.

### Statistical analysis

All statistical analyses were conducted using SPSS statistical package software version 22.0 for Windows (SPSS Inc., Chicago, IL, USA). For continuous variables with normal distribution, data were analyzed with Student’s t-test. Whether variables were normally distributed was tested using the Kolmogorov-Smirnov test. If variables were not normally distributed, independent data were analyzed by means of Mann-Whitney U test while paired data were compared using the Wilcoxon signed rank test. Categorical variables were calculated using Fisher’s exact test or the χ2 test. To assess the power analysis, we used G-power program (version 3.1.9.4). Descriptive statistics are presented as mean ± SD. Differences were regarded as statistically significant at *P* < 0.05.

## Results

**Fig. 1:**
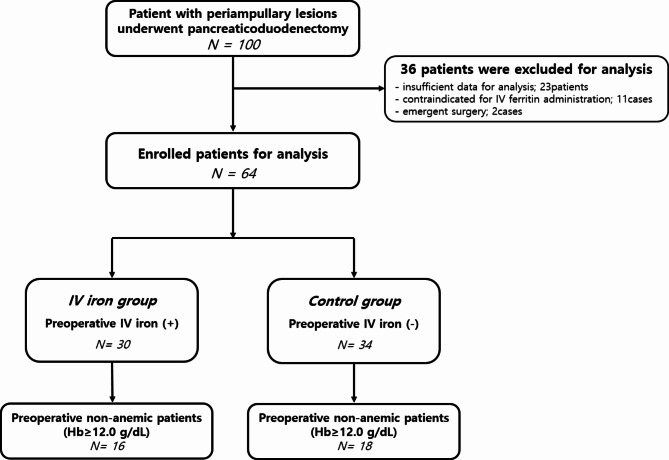
Schematic diagram showing patient enrollment for this study

During the study period, a total of 100 patients underwent PD due to periampullary lesions in our institution. Among them, 36 patients were excluded according to the exclusion criteria: two patients with emergent surgery, 23 patients with insufficient data for analysis, and 11 patients with contraindication for intravenous iron administration. Finally, a total of 64 patients were analyzed for this study, including 30 patients who were administered with intravenous iron preoperatively (46.9%, IV iron group) and 34 patients who had no intravenous iron treatment (53.1%, control group). In enrolled participants, 34 (53.1%) patients were classified as preoperative non-anemic patients (defined as preoperative Hb level above 12.0 g/dl), including 16 (47.1%) patients who underwent intravenous iron treatment preoperatively and 18 (52.9%) patients who had no iron administration. Figure 1 shows a schematic diagram of patient enrollment. Comparative analysis of baseline characteristics between the IV iron group and the control group of participants are presented in Table 1. There was no difference in demographic characteristics such as age, gender, presence of preoperative anemia, preoperative transfusion, and or tumor profiles between the two groups. Patients with preoperative anemia did not receive specific treatment prior to surgery, except for two cases who received blood transfusions preoperatively, according to our institution’s policy which usually do not prescribe specific treatment for anemia unless the hemoglobin level less than 9.0–10.0 g/dL. Table 2 shows results of comparing clinical outcomes of the two groups. There was no significant difference in the incidence of postoperative complications, in-hospital mortality, length of hospitalization, or intensive care unit (ICU) stay. During the study period, 26 (40.6%) patients (13 patients in the IV iron group and 13 patients in the control group, *p = 0.800*) developed postoperative complications, including six patients who had major complications [3 patients in the IV iron group (one case of bile leak treated by percutaneous transhepatic biliary drainage, one case of POPF grade B treated by percutaneous drainage, and one case of delayed gastric emptying with ileus) and 3 patients in the control group (one case of bile leak treated by percutaneous transhepatic biliary drainage and two cases of POPF grade B treated by drainage), *p = 1.000*].

**Table 1:** Comparative analysis of baseline characteristics

Variables	Preoperative IV iron	No IV iron	*p-value*
	*n = 30 (46.9%)*	*n = 34 (53.1%)*	
**Demographics**			
Age (years)	67.5 ± 13.4	69.8 ± 13	*0.502*
Gender (male, %)	10 (33.3)	18 (52.9)	*0.136*
Body mass index (kg/m^− 2^)	23.6 ± 2.3	23.3 ± 2.4	*0.589*
ASA class over III	5 (16.7)	4 (11.8)	*0.723*
Diabetes mellitus	8 (26.7)	5 (14.7)	*0.351*
Hypertension	14 (46.7)	13 (38.2)	*0.613*
History of stroke	3 (10)	2 (5.9)	*0.659*
Preoperative anemia^*^ (%)	14 (46.7)	15 (44.1)	*1.000*
Transfusion before operation	2 (6.7)	0	*0.216*
Use of anticoagulant	7 (23.3)	10 (29.4)	*0.777*
**Tumor location**			
Pancreas	16 (53.3)	16 (47.1)	*0.802*
Duodenum	1 (3.3)	1 (2.9)	*1.000*
Common bile duct	9 (30)	11 (32.4)	*1.000*
Ampulla of Vater	4 (13.3)	5 (14.7)	*1.000*
Others	0	1 (2.9)	*1.000*
**Tumor staging** ^**^			
Stage I	11 (36.7)	13 (38.2)	*1.000*
Stage II	10 (33.3)	13 (38.2)	*0.796*
Stage III	9 (30)	7 (20.6)	*0.405*
**Tumor manifestations**			
Concomitant malignancy	9 (30)	3 (8.8)	*0.052*

^*^Patients with a preoperative hemoglobin level less than 12.0 g/dL were defined as preoperative anemic patients according to the criteria used in several previous studies (17–19)

^**^Cancer stage was evaluated based on the AJCC Cancer Staging Manual, 7th edition (13)

**Table 2:** Comparative analysis of clinical outcomes

Variables		Preoperative IV iron	No IV iron	*p-value*
		*n = 30 (46.9%)*	*n = 34 (53.1%)*	
**Perioperative outcome**				
Operative time (min)		354.6 ± 46	328.3 ± 67.1	*0.071*
Estimated blood loss (ml)		410 ± 450	390 ± 490	*0.799*
Length of ICU stay (day)		1.4 ± 0.7	1.1 ± 0.4	*0.093*
Length of hospital stay (day)		11 ± 6.1	11.5 ± 4.1	*0.750*
Postoperative transfusion		4 (13.3)	9 (26.5)	*0.228*
Postoperative morbidities (%)		13 (43.3)	13 (38.2)	*0.800*
Complication grade^**^ (%)	Grade I/II	10 (33.3)	10 (29.4)	*0.791*
	Grade III/IV/V	3 (10)	3 (8.8)	*1.000*
In hospital mortality (%)		0	1 (2.9)	*1.000*

^**^Postoperative morbidities were reviewed and classified according to the *Clavien-Dindo* classification (14). In complications grading system by *Clavien-Dindo* classification, grade I and II are considered as minor, and grade III to V are defined as major complications

**Fig. 2:**
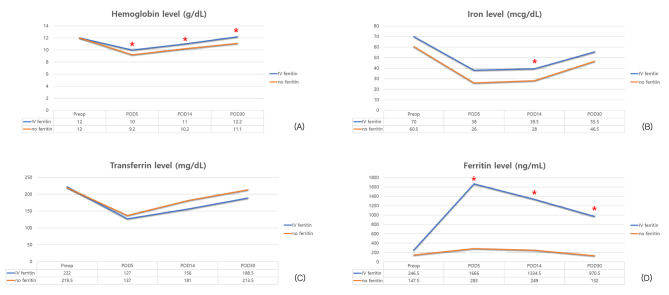
Trends of changes in hematologic profiles such as hemoglobin, iron, transferrin, and ferritin over time in the two groups classified according to preoperative IV iron treatment (IV iron group and control group)

Figure 2 shows trends of changes in iron status parameters (such as hemoglobin, iron, transferrin, and ferritin) over time in both groups (IV iron group and control group). The hemoglobin level decreased immediately after surgery compared to baseline hemoglobin level. It then gradually increased thereafter in both groups. It was significantly higher in the IV iron group from immediately after surgery to later time point. Serum iron level was decreased significantly in both groups after surgery. It maintained until 14 days after surgery, but increased thereafter. The iron level was significantly higher in the IV iron group on day 14 postoperatively. At other observation points, its mean value was higher in the IV iron group, although the difference between the two groups was not statistically significant. Serum transferrin level showed no significant difference between the two groups during the whole observational period. However, ferritin levels were significantly higher in the IV iron group throughout the postoperative period with statistical significance. We performed power analysis using G-power program (version 3.1.9.4) based on the setting 0.96 of effect size, 0.05 of alpha, and 34 and 30 of sample size in each group. The effect size was calculated based on the mean value 11.3 and 10.2 of hemoglobin level measured at postoperative 2 weeks and 1.05 and 1.23 of standard deviation in two groups. As a result, the power value was measured as 0.965 and the Beta was calculated as 0.035.

**Fig. 3:**
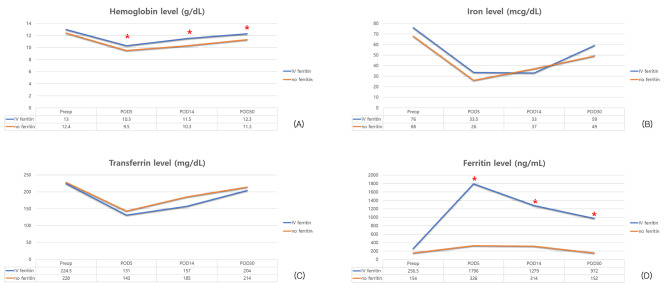
Trends of changes in hematologic profiles such as hemoglobin, iron, transferrin, and ferritin levels of preoperative non-anemic patients over time in the two groups classified according to preoperative IV iron treatment (IV iron group and control group)

In addition, a subgroup analysis was performed for clinical outcomes and changes in hematological profiles of 34 preoperative non-anemic patients following IV iron treatment. Demographic characteristics were similar between the two groups (Table 3). In the comparison of clinical outcomes (Table 4), the length of ICU stay was significantly shorter in the IV iron group. Postoperative morbidities were developed in 14 cases (9 cases in IV iron group and 5 cases in control group, *p = 0.163*). Major complication was observed in three cases (two cases in the IV iron group included one case of grade B POPF and another case of intraabdominal abscess treated by radiologic intervention, and one case in the control group that was developed grade B POPF). Figure 3 shows comparison of changes in hematologic profiles over time in preoperative non-anemic patients. All hematologic profiles except for ferritin level were decreased immediately after surgery and gradually increased at 5 days after operation in the control group. However, they failed to return to preoperative levels by 30 days postoperatively. In the case of ferritin level, it was maintained at similar to the preoperative level. On the contrary, the level of hemoglobin was recovered to the preoperative level on the 30th day after surgery in the IV iron group. Thereafter, hemoglobin levels were significantly higher in the IV iron group throughout the postoperative period. In particular, when 12.0 g/dL of Hb was used as the standard for preoperative anemia, mean Hb levels decreased to less than 12.0 g/dL in both groups immediately after surgery. However, in the IV iron group, it recovered to 12.0 g/dL or higher from 14 days postoperatively and maintained thereafter, whereas it did not reach 12.0 g/dL of Hb level in the control group by 30 days postoperatively. Regarding serum iron, its mean value was higher in the IV iron group without showing statistically significant difference at 30 days postoperatively (59 mcg/dL in the IV iron group and 49 mcg/dL in the control group, *p = 0.249*). Ferritin levels remained significantly higher in the IV iron group throughout the postoperative observational period, as same as shown in all patients.

**Table 3:** Comparative analysis of baseline characteristics in preoperative non-anemic patients who had preoperative Hb level above 12.0 g/dL

Variables	Preoperative IV iron	No IV iron	*p-value*
	*n = 16 (47.1%)*	*n = 18 (52.9%)*	
**Demographics**			
Age (years)	65.3 ± 9.2	68.9 ± 11	*0.306*
Gender (male, %)	14 (87.5)	10 (55.6)	*0.063*
Body mass index (kg/m^− 2^)	24.3 ± 2	23.6 ± 2.5	*0.349*
ASA class over III	3 (18.8)	2 (11.1)	*0.648*
Diabetes mellitus	3 (18.8)	3 (16.7)	*1.000*
Hypertension	7 (43.8)	8 (44.4)	*1.000*
History of stroke	0	2 (11.1)	*0.487*
Use of anticoagulant	5 (31.2)	6 (33.3)	*1.000*
**Tumor location**			
Pancreas	8 (50)	10 (55.6)	*1.000*
Duodenum	1 (6.2)	0	*0.471*
Common bile duct	4 (25)	6 (33.3)	*0.715*
Ampulla of Vater	3 (18.8)	1 (5.6)	*0.323*
**Tumor staging** ^**^			
Stage I	8 (50)	10 (55.6)	*1.000*
Stage II	4 (25)	6 (33.3)	*0.715*
Stage III	4 (25)	1 (5.6)	*0.164*
**Tumor manifestations**			
Concomitant malignancy	4 (25)	2 (11.1)	*0.387*

^*^Cancer stage was evaluated based on the AJCC Cancer Staging Manual, 7th edition (13)

**Table 4:** Comparative analysis of clinical outcomes in preoperative non-anemic patients who had preoperative Hb level above 12.0 g/dL

Variables		Preoperative IV iron	No IV iron	*p-value*
		*n = 16 (47.1%)*	*n = 18 (52.9%)*	
**Perioperative outcome**				
Operative time (min)		362.7 ± 45.4	331.8 ± 49.3	*0.072*
Estimated blood loss (ml)		390 ± 230	310 ± 170	*0.249*
Length of ICU stay (day)		1 ± 0.1	1.7 ± 0.9	*0.012*
Length of hospital stay (day)		12.9 ± 7.9	11.1 ± 4.8	*0.445*
Postoperative transfusion		0	2 (11.1)	*0.487*
Postoperative morbidities (%)		9 (56.2)	5 (27.8)	*0.163*
Complication grade^*^ (%)	Grade I/II	7 (43.8)	4 (22.2)	*0.274*
	Grade III/IV/V	2 (12.5)	1 (5.6)	*0.591*
In hospital mortality (%)		0	0	

^*^Postoperative morbidities were reviewed and classified according to the *Clavien-Dindo* classification (14). In complications grading system by *Clavien-Dindo* classification, grade I and II are considered as minor, and grade III to V are defined as major complications

## Discussion

We aimed to evaluate overall changes in hematologic profiles of patients who underwent PD, and investigate the effect of preoperative IV iron treatment on recovery of anemia after surgery. Our results revealed that patients who underwent PD had similar patterns of postoperative changes in hematologic profiles regardless of the presence of a preoperative anemia. Values of hematologic parameters were significantly decreased immediately after surgery. They showed a gradual recovery during the observation period. Hemoglobin and iron levels were significantly higher in patients receiving preoperative IV iron than those in patients without receiving preoperative IV iron. The same result was observed not only in the group with preoperative anemia, but also in the group with a serum hemoglobin value of 12 or higher.

In addition to etiologic factors (such as bleeding, malignancy, and chronic disease) of surgery, surgically induced inflammatory responses can promote the synthesis of hepcidin and reduce iron bioavailability. It can inhibit iron absorption from the gastrointestinal tract and reduce the release of stored iron [20]. The level of ferritin, an acute phase reactant, may temporarily change under the condition of post-operative inflammation. An insufficient red blood cell production can result in IDA when stored iron is depleted. These phenomena are evidently observed in results of our study, especially more pronounced in the control group without receiving preoperative IV iron. Hemoglobin and iron levels were decreased after surgery. After that, iron depletion continued and ferritin levels maintained without showing a recovery, whereas transferrin levels were increased. On the other hand, patients who received preoperative IV iron did not show a decrease in ferritin level, but rather maintained high levels during the observation period, although hemoglobin and iron levels were similarly decreased immediately after surgery. At two weeks after surgery, hemoglobin and iron levels were significantly higher in patients who received preoperative IV iron than in patients who did not receive preoperative IV iron. Therefore, preoperative IV iron management might be helpful for preventing IDA caused by postoperative physiological changes and iron depletion.

In fact, several studies have previously reported that preoperative iron supplementation is helpful in the prevention of postoperative IDA [5–8]. However, results of this study showed effects of preoperative IV iron treatment in all patients who underwent PD with or without preoperative IDA. Similar to patients diagnosed with preoperative IDA, the hemoglobin level was significantly decreased immediately after surgery in normal patients without IDA prior to surgery. The preoperative IV iron group also showed a significantly faster recovery of hemoglobin level. These results might be due to characteristics of the PD surgery itself. PD is a complex and difficult surgical procedure that requires resection of the entire duodenum together with the head of the pancreas, proximal jejunum, distal common bile duct, and the gastric antrum sometimes. Considering that most iron is absorbed into the body through the duodenum and proximal portion of jejunum, in the case of PD with total removal of those organs during surgery, a significant change occurs in the normal iron absorption process after surgery. Thus, the risk of IDA is increased after PD due to an absolute lack of iron absorption from the intestinal tract. This suggests that even non-specific patients without preoperative IDA can be high-risk candidates for developing postoperative IDA due to physiological characteristics of the surgical technique of PD itself. In addition, in the case of cancer surgery, the risk of postoperative anemia may increase due to the high invasiveness caused by resection of adjacent organs and radical lymph node dissection. Prolonged fasting is common after PD due to concerns about anastomotic leakage, which can lead to insufficient iron supplementation for a long time. Since patients after PD become vulnerable to IDA regardless of the presence of preoperative anemia, preoperative IV iron treatment should be considered for all patients who are planned to undergo PD.

Since postoperative IDA is well known to be associated with poor prognosis of clinical outcomes, an active intervention by clinicians for the prevention of IDA before surgery is important. Although it is true that both pros and cons about the actual clinical effect of intravenous iron treatment in anemic patients have been reported in previous studies, there is no major disagreement about whether the presence of anemia has a negative impact on the clinical outcomes of patients after surgery. *Munoz, M. et al.* [21] reported that perioperative anemia increases postoperative morbidity and mortality, and *Fowler et al.* [2] reported that preoperative anemia is associated with an increased risk of blood transfusion, in-hospital complications, delayed hospital discharge, and poor recovery in patients undergoing major elective surgery. Epidemiological studies have reported that anemia is associated with increased rates of postoperative morbidity and mortality [22, 23]. Considering these negative effects of anemia, the authors expect to improve clinical outcomes through the prevention and treatment of anemia by promoting the recovery of the patient’s hematological profile after surgery through active preoperative intervention such as intravenous iron treatment. Actually, *Philip et al.* [24] reported that IV iron treatment can significantly increase hemoglobin level in iron deficiency anemic patients undergoing colorectal surgery, with reduction in red cell transfusions. Also, IV iron was reported as more efficacious at improving quality of life scores than oral iron in surgical anemic patients using SD36, EuroQoL 5-dimension 5-level and Functional Assessment of Cancer Therapy – Anaemia questionnaires [6]. In a study of *Khalafallah* et al. the group receiving IV iron treatment showed shortened length of hospital stay probably due to fewer postoperative blood transfusions or infections [12]. In our results, the length of ICU stay was also significantly shortened in the IV iron group having no preoperative anemia than in the control group. Due to limitations in the design of this study, it is difficult to determine whether this difference in ICU stay was due to the effect of preoperative IV iron treatment rather than other baseline characteristics and confounders, such as burden of ICU care costs or transfer to another hospital during treatment. It was impossible to statistically analyze the effects due to such costs burden or missing data due to insufficient data. However, preoperative IV iron treatment for patients undergoing PD not only can help prevent postoperative anemia and early recovery of hematologic profiles, but also can improve postoperative clinical outcomes. IDA causes fatigue, inhibition of wound healing, and loss of appetite in cancer patients who have undergone major abdominal operations [6], which ultimately leads to a delay in physical recovery after surgery or the initiation of appropriate adjuvant treatment such as chemotherapy or radiation therapy. It can reduce the compliance to treatment and eventually lead to discontinuation or impede the effectiveness of these treatments. Although this study did not serially observe changes in long-term postoperative outcome with preoperative IV iron treatment, for patients with malignant tumors for whom the importance of follow-up anticancer treatment that should be administered continuously after surgery is crucial for long-term survival, the prevention of developing IDA after surgery through preoperative IV iron treatment will have significant significance in improving oncologic outcomes. Additionally, IDA that occurs after surgery would be more proper to prevent before it occurs than to treat it through blood transfusions after it occurs. As seen in many previous studies [25–27], allogeneic blood transfusion has a risk of transfusion-transmitted infections. Emerging pathogens can infect the blood supply [28]. From a non-infectious aspect, it can cause immune-mediated acute transfusion reactions [29]. In cancer patients, blood transfusion can have a negative oncologic effect such as promoting a protumor state that might cause cancer recurrence [7]. In addition, preventing postoperative IDA through preoperative interventions may reduce the cost burden of treating postoperative IDA using blood transfusions or other supplements to enhance iron supply. Considering the high cost of transfusion products such as red blood cell packs, it would certainly be more effective to prevent IDA before it occurs rather than to treat it after it occurs. Therefore, it is necessary to avoid unnecessary blood transfusions after surgery by actively preventing the occurrence of postoperative IDA before the surgery through iron supplement. However, there are studies such as PREVENTT trial [8] that reported negative results regarding the actual outcome improvement effect of perioperative iron treatment. Thus, a well-designed large-scale study is still needed to confirm whether the theoretical advantages of preoperative intravenous iron treatment and the advantages reported in previous studies can actually lead to improved clinical outcomes in all patients.

Despite these interesting results, our study has inevitable limitations that warrant caution when interpreting results. First, due to its prospective cohort design of study, there might be a selection bias of enrolled patients with different characteristics in disease or demographics by phase. In addition, participants might have different treatment protocols that might have changed slightly over time. However, a single, same clinician has been managing all critically ill patients after surgery at our intensive care unit since 2016. Therefore, the treatment strategy remained relatively constant during the study period and the treatment principle for anemia patients, the focus of this study, remained constant without change. Therefore, potential treatment bias was minimized. Additionally, we did not measure or analyze some laboratory profiles such as hepcidin or transferrin saturation known to be potential biomarkers for responses to iron treatment. Finally, our study included a relatively small number of cases in a single institution and we failed to confirm the significant differences in clinical outcomes such as complications or mortality between two groups. In particular, in interpreting the results derived from this study, the positive effects that preoperative IV iron treatment can have clinically were interpreted with limited possibilities. Additionally, since we analyzed the changes of hematologic profiles during 30 days after surgery, we could not assess the changes after that and failed to confirm the lasting effect of IV iron treatment after 30 days. In order to overcome these limitations, a prospective randomized-controlled trial with a larger number of participants should be conducted in the near future along with analysis of more diverse hematologic profiles. However, to the best of our knowledge, this was the first study that analyzed the pattern of hematologic profiles before and after surgery in patients undergoing PD. In particular, it compared patterns of changes in hematological values according to IV iron treatment before surgery even in non-specific patients without preoperative anemia. Additionally, the effectiveness of postoperative iron supplements in recovering the hematological profile of patients after surgery have been reported in many studies. But especially in the case of surgeries that inevitably involve complex anastomosis sites, such as PD, the incidence of postoperative complications is relatively high, so depending on the patient’s condition, it may be difficult to administer intravenous iron treatment after surgery. Also, considering complications such as ileus or decreased bowel movements, which are common complications after abdominal surgery, it is thought that the postoperative use of iron supplements with concerns about constipation may have some limitations. Considering these points, it is expected that there will be a differentiated advantage of IV iron treatment performed before surgery, especially when patients scheduled to undergo elective surgery are in relatively good physical condition. Given that there is currently insufficient evidence to establish IV iron treatment for patients undergoing PD, our findings suggest that preoperative IV iron administration can be an effective treatment option for all patients with PD for the prevention and early recovery of postoperative IDA.

In conclusion, most patients undergoing PD showed significantly decreased hemoglobin levels after surgery regardless of the presence of preoperative anemia. Preoperative IV iron treatment might be effective in facilitating recovery of hematologic profiles of patients during the recovery period after PD regardless of the presence of preoperative IDA, consequently resulting in prevention of postoperative IDA. Further study with large samples are needed to determine the prognostic effects of preoperative IV iron treatment on postoperative complications.

### Electronic supplementary material

Below is the link to the electronic supplementary material.

Supplementary Material 1

Supplementary Material 2

## Data Availability

The research data that support the findings of this study are available from the corresponding author upon reasonable request. These research data are not publicly available due to privacy or ethical restrictions.

## Abbreviations

IDA: iron deficiency anemia
PD: pancreaticoduodenectomy
IV: intravenous
Hb: hemoglobin
POPF: postoperative pancreatic fistula
ICU: intensive care unit
