# Supplementary material for: What we should consider to facilitate recovery of the hematological profile in all patients after pancreaticoduodenectomy: the role of preoperative intravenous iron treatment
Source: BMC Surg. 2023 Oct 12;23:308. doi: 10.1186/s12893-023-02217-x (PMC10571369; doi:10.1186/s12893-023-02217-x)
Supplement: Supplementary file 1 — Supplementary Material 1 [file 12893_2023_2217_MOESM1_ESM.docx]

< Appendix 1. Operative techniques of pancreaticoduodenectomy >

The operative procedures for pancreaticoduodenectomy were same as previously reported in *Lee* et al. [29] and were similar to those for conventional open pancreaticoduodenectomy. In cases of malignancy, radical lymph node dissection was performed using ultrasonic coagulating shears, and the range of dissection included the lymphoid tissues around the peripancreatic area, the inferior vena cava, the aorta, the common hepatic artery, the proximal 0.5–1 cm of the celiac axis and portal vein, and the right side of the superior mesenteric artery. We skeletonized these structures individually up to the porta hepatis. We have selectively performed pancreaticogastrostomy (PG) for patients who have pancreas with extremely small pancreatic duct and bulky pancreas with soft texture. Otherwise, duct-tomucosa pancreaticojejunostomy (PJ) was performed. The procedure was performed with steps similar to those used in the open approach for a standard PD. Mobilization and dissection of the duodenum, bile duct, and jejunum were performed sequentially. The neck of the pancreas and the uncinate process were then dissected. The surgeon moved from the left to the right side of the patient as needed to perform the operation. For cases of small pancreatic duct with bulk and soft texture, PG method was adapted for pancreaticoenteric anastomosis. First, the proximal jejunum was pulled up in a retrocolic fashion through the mesentery of transverse colon, and the end to side choledochojejunostomy (CJ) was performed intracorporeally before PG. After the completion of CJ anastomosis, we made longitudinal mini-laparotomy incision about 5 cm in length at the epigastrium over the pancreatic stump directly and removed the specimen through it. As the surgeon performed PG through an anterior gastrostomy looking directly down on the stump of the pancreas, a longer incision was unnecessary. were able to perform PG safely. On the other hand, PJ was performed in a totally laparoscopic method for cases with hard pancreas texture or with dilated pancreatic duct. For PJ, duct-to-mucosa anastomosis was performed laparoscopically. Interrupted sutures between the pancreatic parenchyma and the jejunal seromuscularis were placed in the outer layer using absorbable sutures (Vicryl #3-0). A small hole compatible with the caliber of the pancreatic duct was made in the jejunal wall using electrocautery. The pancreatic duct was then directly sutured to the jejunal mucosa with short internal stent and absorbable sutures (Monosyn #4-0 or #5-0) using 4–6 interrupted stiches. Duodenojejunostomy (DJ) was then performed in an antecolic, end-to-side fashion through minilaparotomy site (in PG cases) or umbilical trocar site with a minimal extension (in PJ cases) using a hand-sewn technique extracorporeally. After reconstruction of the digestive tract, the specimen was extracted using a retrieval bag through an umbilical trocar site. Two closed suction drains were used (one for the pancreaticoenteric anastomosis site through the left-sided 5-mm port incision while the other for the CJ site through the right flank port site).
