# Supplementary material for: What we should consider to facilitate recovery of the hematological profile in all patients after pancreaticoduodenectomy: the role of preoperative intravenous iron treatment
Source: BMC Surg. 2023 Oct 12;23:308. doi: 10.1186/s12893-023-02217-x (PMC10571369; doi:10.1186/s12893-023-02217-x)
Supplement: Supplementary file 2 — Supplementary Material 2 [file 12893_2023_2217_MOESM2_ESM.docx]

< Appendix 2. Definitions of complications >

| **Complication** | **Definition** |
| --- | --- |
| **Postoperative pancreatic fistula (POPF)** |  |
| Grade A POPF | Clinically stable and requires no treatment or admission, and therefore it is not considered as a morbidity |
| Grade B POPF | Fluid collection on computed tomography scan and may require treatment and/or readmission |
| Grade C POPF | Clinically unstable and requires treatment or reoperation, and sepsis or infection may be present |
| **Surgical site infection (SSI)** |  |
| Superficial incisional SSI | An infection involving only skin or subcutaneous tissue at the site of the incision |
| Deep incisional SSI | An infection which appeared to be related to the operation and involved deep soft tissues |
| Organ-occupying SSI | An infection seemed to be related to the operation and involved any part of the anatomy other than the operative incision |
| **Pneumonia** | Chest radiographs postoperatively develop with new infiltrates, consolidation or cavitation. And patients also met at least  one of the following A-C, and at least two of the following a-d. |
| A | Fever (> 38℃) with no other recognized cause |
| B | Leucopenia (white cell count < 4 × 109 L−1) or leukocytosis (white cell count > 12 × 109 L−1) |
| C | For adults > 70 years old, altered mental status with no other recognized cause |
| a | New onset of purulent sputum or a change in character of the sputum, or increased respiratory secretions |
| b | New onset or worsening cough, or dyspnea, or tachypnea |
| c | Rales or bronchial breath sounds |
| d | Worsening gas exchange (hypoxemia, increased oxygen requirement, increased ventilator demand) |
